# Mythical Snakes

**Published:** 1892-02

**Authors:** 


### MYTHICAL SNAKES.

The cause of persons whose nerves are excited by protracted and excessive use of stimulants seeing the shapes of animals passing before them is not due wholly to imagination. In fact, the fancy only operates to induce a belief that what is seen is alive and hideous.

The eyeball is covered by a network of veins, ordinarily so small that they do not intrude themselves visibly in the path of the light that enters the sight, but in the couse of some diseases these veins are frequently congested and swollen to such a size as to become visible, and when this happens the effect generally is to appear as if there were an object of considerable size at a distance from the eye.

Of course, this vein is generally long, thin and sinuous like a serpent, and the figure seen is frequently startlingly like a snake. That they seem to live is due to the fact that they are often not in perfect line with the direct front of sight. They are either to the side, up or down from the focus, therefore, when discovered, the victim naturally turns his eyes toward the effect, and the effect, of course, moves away.

The eye follows, and thus a continuous and realistic motion is got. Now, if the eye be turned to the front again quickly, it will see another snake, which, if watched will glide away in the same manner. The writer of this is afflicted by malarial disease, and after his eyes are thus congested many strange shapes and clouds pass within his vision, which, if he were in a state of nervous collapse, might easily be all that are seen by those suffering from delirium tremens.

